# Is the Code of Ethics a Failure?

**Published:** 1889-05

**Authors:** 


### EDITORIALS AND MISCELLANEOUS.

IS THE CODE OF ETHICS A FAILURE?—

"Not obvious, not obtrusive, but retired,
The more desirable."

The vanity of some medical men is in flagrant violation of the above maxim. When we hear of or read the actions of those who would have us believe that they stand at the head of the profession, or of the teaching corps of this country, we feel like snatching the Code of Ethics and throwing it in the fire, for it is not worth the paper it is written on.

What avails it that the young graduate on the threshold of the profession has been taught in his Alma Mater that the condition "sine qua non" to be in good standing, and to be admitted in both the local and State medical associations, is to strictly conform to the Code, when there hardly passes a week but the *irrepressible* reporter is very *conveniently* made responsible for an interview with one of those would be medical Nabobs?

It does not take a grain of intuition to fathom the depths of these designing schemes. For my part I have no more respect for that species of charlatanry than I have for him who proposes to cure all the ills human flesh is heir to with his lebenswecker, or to cure piles on sight without knife, ligatures or even touching them. For it makes no differance whether a man chronicles his "*Specialty*" in the lay journals or puffs himself up through the press with the regularity of menstrual periods, the intent is evidently and palpably the same. In either case *it is advertising*, with this difference, the one is frank and open and comparatively honest; the other is designedly hidden and of doubtful morality.

A consultation, an *unsolicited* or *unsought for* lecture, a charitable contribution, a dinner speech, a biographical sketch, a touching eulogy, a call from a distance, etc., etc. are very convenient topics for advertising nowadays, and as sure *to hit* as the multiform schemes that give publicity and celebrity to Warner's Safe Liver Cure.

To me this is perfectly sickening. I have written these remarks not to propose or even suggest a remedy. There are so many ways of evading the rules of the Code; so many blind tigers, and as Wilkins says: "There is no work impossible to these contrivances."

I think it proper however, to expose such methods. They are reprehensible. The fact that they are resorted to by men who have acquired some reputation in the profession does not secure them against censure. On the contrary, such men should, like Cæsar's wife, be above reproach and teach their younger colleagues who yet have to struggle for the obtaining of a practice and the securing of a competency, to respect and love their profession and those who should be by their experience, knowledge, and honorable dealings, looked up to as model physicians. E. V. G.

